# A Machine Learning Model for Predicting the Risk of Readmission in Community-Acquired Pneumonia

**DOI:** 10.7759/cureus.29791

**Published:** 2022-09-30

**Authors:** Mohammed D Aldhoayan, Hazza Alghamdi, Afnan Khayat, Rajkumar Rajendram

**Affiliations:** 1 Health Affairs, King Abdulaziz Medical City, Riyadh, SAU; 2 Health Informatics, King Saud Bin Abdulaziz University for Health Sciences, Riyadh, SAU; 3 Health Information Technology Affairs, King Faisal Specialist Hospital & Research Centre, Riyadh, SAU; 4 College of Medicine, King Saud Bin Abdulaziz University for Health Sciences, Riyadh, SAU; 5 Medicine, King Abdulaziz Medical City, Riyadh, SAU

**Keywords:** artificial intelligence, readmission, machine learning, prediction model, pneumonia

## Abstract

Background

Pneumonia is a common respiratory infection that affects all ages, with a higher rate anticipated as age increases. It is a disease that impacts patient health and the economy of the healthcare institution. Therefore, machine learning methods have been used to guide clinical judgment in disease conditions and can recognize patterns based on patient data. This study aims to develop a prediction model for the readmission risk within 30 days of patient discharge after the management of community-acquired pneumonia (CAP).

Methodology

Univariate and multivariate logistic regression were used to identify the statistically significant factors that are associated with the readmission of patients with CAP. Multiple machine learning models were used to predict the readmission of CAP patients within 30 days by conducting a retrospective observational study on patient data. The dataset was obtained from the Hospital Information System of a tertiary healthcare organization across Saudi Arabia. The study included all patients diagnosed with CAP from 2016 until the end of 2018.

Results

The collected data included 8,690 admission records related to CAP for 5,776 patients (2,965 males, 2,811 females). The results of the analysis showed that patient age, heart rate, respiratory rate, medication count, and the number of comorbidities were significantly associated with the odds of being readmitted. All other variables showed no significant effect. We ran four algorithms to create the model on our data. The decision tree gave high accuracy of 83%, while support vector machine (SVM), random forest (RF), and logistic regression provided better accuracy of 90%. However, because the dataset was unbalanced, the precision and recall for readmission were zero for all models except the decision tree with 16% and 18%, respectively. By applying the Synthetic Minority Oversampling TEchnique technique to balance the training dataset, the results did not change significantly; the highest precision achieved was 16% in the SVM model. RF achieved the highest recall with 45%, but without any advantage to this model because the accuracy was reduced to 65%.

Conclusions

Pneumonia is an infectious disease with major health and economic complications. We identified that less than 10% of patients were readmitted for CAP after discharge; in addition, we identified significant predictors. However, our study did not have enough data to develop a proper machine learning prediction model for the risk of readmission.

## Introduction

The Centers for Disease Control and Prevention (CDC) has defined pneumonia as “a lung infection that can cause mild to severe illnesses in people of all ages.” As a worldwide infectious disease, pneumonia is the leading cause of death in children less than five years of age [1]. In the United States, pneumonia results in around one million admissions yearly, and up to 20% of these admissions require intensive care, leading to more than 50,000 deaths. Pneumonia is also responsible for almost 140,000 hospital readmissions costing up to 10 billion US Dollars [2]. In a study that included patients discharged after being treated for pneumonia, almost 7.3% were readmitted in less than a month [3]. However, the average rate of readmission increased to 18.5% in 2012 [4]. With the increase in readmission rates, Medicare and Medicaid Services (CMS) recognized the financial impact of hospital readmissions and started to assess the event by introducing readmission adjustment factors for hospital reimbursement [5]. The focus on improving the readmission rate was not only an incentive raised financially but also to improve the quality of patient care [4].

The impact of readmission has raised the awareness to investigate its causes and risk factors. Many studies have been conducted to investigate these factors. Hebert et al. found that the risk factors identified can vary from pneumonia-related (35.8%) to comorbidity-related reasons [3]. While others recognized instability on discharge and treatment failure as possible causes [6]. A systematic review that studied social factors as possible readmission risk factors concluded that age (elderly), race (non-whites), education (low), unemployment, and low income were associated with a higher risk of hospital readmission [7]. In another study investigating the history of readmitted patients, 50.2% of the readmitted patients had a medical discharge with no follow-up visits until readmission. It should be noted that the readmission length of stay was reported to be almost 0.6 days longer than the usual length of stay [8]. Epstein et al. also highlighted a proportional relationship between overall hospital admission and readmission rate [9].

Machine learning methods have been used to guide clinical judgment in gray areas as they can predict a pattern based on data and help decision-making. In 1959, machine learning was defined as a “Field of study that gives computers the ability to learn without being explicitly programmed.” Many prediction algorithms have been proposed and successfully used in healthcare, such as support vector machine (SVM), decision tree (DT), and logistic regression, and have been tested in many medical conditions such as diabetes among other conditions [10].

The importance of creating prediction models for readmission is to overcome all previously mentioned complications. Due to the difficulties in predicting readmission, the model can guide healthcare providers (HCPs) in improving/supporting clinical decision-making during healthcare episodes while considering the risk of readmission [4]. As a result, the CMS has started readmission reduction program initiatives to push healthcare systems to find new innovative ways to predict and tackle these issues [11]. Multiple researchers have developed predictive models to identify patients who are more likely to be readmitted [12].

In 2016, a systematic review identified all used models and described their performances. The study concluded that only a few validated models predict the risk of readmission of pneumonia patients, the published models were moderately acceptable, and that any future model should have additional parameters to improve the prediction accuracy, or as suggested by O'Brien et al. to include all available patient care information if possible [13,14].

Another study aimed at predicting readmissions in general not specifically to pneumonia at King Abdulaziz Medical City (KAMC) in Riyadh, Saudi Arabia, used neural network (NN), SVM, rules-based techniques, and C4.5 DT and concluded that the models achieved higher accuracy levels when the prediction was designed for readmission within 15 days only. Furthermore, the study suggested that SVM was the best model. Of all the variables used in the training set, the visit history, lab tests, and patient age had more weight in the prediction than other variables in addition to the length of stay before discharge [15].

Furthermore, other researchers have developed logistic regression models with stepwise removal for the prediction that showed better prediction models [4]. Other studies have not disclosed the used prediction models and simply reported using logistic regression models for the prediction without further details [6]. Thus, this study aims to build a prediction model for the readmission risk within 30 days of discharge in patients discharged after community-acquired pneumonia (CAP) management. The main contributions of this study are testing previously used models while adding more parameters to improve the prediction model. The parameters are based on literature recommendations and data availability. In addition, we aimed to evaluate the quality of the Hospital Information System (HIS) datasets and use the model for feature selection from the dataset to identify risk factors and variations between subgroups.

## Materials and methods

Data collection

Multiple machine learning models were used to predict readmission of CAP patients within 30 days of diagnosis by conducting a retrospective observational study on patient data. The dataset was obtained from multiple tertiary healthcare organizations in Saudi Arabia. All patients diagnosed with CAP from 2016 until the end of 2018 were included. We excluded pediatric patients who were less than 18 years old or patients who died within 30 days of discharge. Ethical approval was obtained from the Internal Review Board (IRB) of King Abdullah International Medical Research Center because the acquired data were de-identified.

Data pre-processing

The raw data were segregated into multiple datasets. The datasets obtained contained different attributes, including the history of pneumonia admissions, vital signs, radiology, laboratory, medication list, and comorbidities. For modeling, different data transformation and cleaning processes were conducted on the dataset. The length of stay (LOS) attribute was changed for patients discharged in less than a day to 1. The age attribute was derived from the date of birth and the admission date. All comorbidities, medications, radiology examinations, and lab results were transformed to dummy attributes with the values 1 and 0 for each admission. Attributes with missing values were filled with the median. Lastly, a new attribute was also derived to represent the class labels (readmission within 30 days of discharge and no readmission) as 0 and 1. Python coding language and Scikit-learn machine learning package were used for dataset cleaning and modeling [16].

Data analysis

Univariate analyses were deployed using logistic regression to study the significance of the relationship between each factor and the readmission. All significant variables with a p-value of less than 0.05 were then passed to multivariate logistic regression, and the odds ratios were calculated to determine the magnitude of the relationship. Furthermore, various classic and modern machine learning models have been reported in the literature. In this study, to predict readmission within 30 days, we experimented with several algorithms using predictive analytics. The first model was developed using RF with a maximum depth of two levels and 100 estimators [17]. Second, a logistic regression (LR, aka logit, MaxEnt) classifier was developed that implemented regularized logistic regression using the “liblinear” solver [18]. The third was developed using DTs with a maximum depth of two levels [19]. The fourth was the SVM classifier [20].

Model evaluation and validation

A cross-validation technique was used by splitting the dataset into 70% of the dataset for training and 30% for testing. To measure the performance of the model, precision, recall, f1-score, and accuracy metrics were used to measure both results, readmitted and not readmitted. These results were calculated for both labels in Python using the Scikit-learn library [16].

## Results

The collected data included 8,057 admission records related to pneumonia for 5,776 patients, including 2,965 males and 2,811 females. Patients were located at six different hospitals under the Ministry of National Guard Health Affairs. Of these admissions, only 791 admissions were followed by a readmission within 30 days, with a percentage of 9.1% of the total number of admissions included in the dataset. The minimum age was 18, and the maximum age was 119, but more than 60% of the patients were older than 66 years old with a median of 70 years (Table 1).

**Table 1:** Admission characteristics. LOS: length of stay

Demographic		Number	Percentage
Gender	Male	4,187	51.2%
	Female	3,870-	47.9%
Age (years_	18–40	888	11%
	41–65	2,023	25%
	66–90	4,648	57.6%
	91–120	498	6.2%
LOS (days)	0–7	4,583	56.9%
	8–14	1,692	21%
	15–29	1,054	13%
	>30	737	9%

Analyses

The first step in our analysis was the univariate logistic regression with each of the independent variables. The results indicated that all the variables were significantly affecting the readmission probability (Table 2).

**Table 2:** Univariate analysis. SD: standard deviation; OR: odds ratio; 95% CI: 95% confidence interval; LOS: length of stay; BP: blood pressure

Predictive attribute	Readmitted (n = 11,286)		Not readmitted (n = 66,620)		Univariate analysis		
	Mean	SD	Mean	SD	OR	(95% CI)	P-value
Age at the event (years)	19.40	69.92	18.54	67.42	0.97	(0.97-0.97)	<0.001
LOS (days)	22.27	14.39	38.44	14.46	0.88	(0.88-0.89)	<0.001
Gender	49%	0.60	50%	0.51	0.13	(0.12-0.14)	<0.001
Temperature	13.28	31.00	14.50	29.63	0.94	(0.94-0.94)	<0.001
Systolic BP	27.77	118.24	30.54	118.29	0.98	(0.98-0.98)	<0.001
Diastolic BP	14.36	62.15	16.02	62.02	0.97	(0.97-0.97)	<0.001
Heart rate	18.17	82.36	18.30	81.71	0.97	(0.97-0.98)	<0.001
Respiratory rate	7.57	18.73	6.68	19.36	0.90	(0.90-0.90)	<0.001
Lab and radiology count	0.39	0.10	0.45	0.12	0.16	(0.13-0.20)	<0.001
Medication count	1.35	0.29	1.84	0.45	0.57	(0.53-0.61)	<0.001
Comorbidity count	2.51	3.41	2.50	2.67	0.60	(0.58-0.61)	<0.001

Subsequently, a multivariate analysis that included all significant factors from the univariate analysis was conducted. The results indicated that keeping other variables constant, the odds of being readmitted were decreased by 1% for each unit increase in age. The odds of being readmitted were 1.01 times higher for every extra temperature unit. The odds of being readmitted decreased by 1% for each unit increase in heart rate. The odds of being readmitted decreased by 2% for each unit increase in the respiratory rate. The odds of being readmitted decreased by 7% for every extra prescribed medication. The odds of being readmitted were 1.08 times higher for every extra comorbidity that the patient had. All other variables showed no significant effect (Table 3).

**Table 3:** Multivariate analysis. OR: odds ratio; 95% CI: 95% confidence interval; LOS: length of stay; BP: blood pressure

Attribute	OR	OR (95% CI)	P>|z|
Age at the event	0.99	(0.98-0.99)	<0.001
LOS	1	(1.00-1.00)	0.52
Gender	1.15	(1.00-1.33)	0.05
Temperature	1.01	(1.00-1.01)	0.01
Systolic BP	1	(0.99-1.01)	0.96
Diastolic BP	0.99	(0.98-1.00)	0.05
Heart rate	0.99	(0.99-1.00)	<0.001
Respiratory rate	0.98	(0.97-0.99)	<0.001
Lab and radiology count	0.85	(0.70-1.03)	0.10
Medication count	0.93	(0.88-0.98)	0.01
Comorbidity count	1.08	(1.06-1.11)	<0.001

For building the prediction model, four different machine learning algorithms were used. DT provided high accuracy of 83%, while SVM, RF, and logistic regression achieved better accuracy of 90%. Because the dataset was unbalanced, the precision and recall for readmission were zero for all models except the DT with 16% and 18%, respectively. On applying the Synthetic Minority Oversampling TEchnique (SMOTE) to balance the training dataset, the results did not change considerably, and the highest precision achieved was 16% in the SVM model [21]. The highest recall was achieved by RF at 45% but without any advantage to this model because the accuracy had reduced to 65%. To understand the impact of adding different attributes included in the dataset and to test whether the deletion would have a positive or negative impact on the model, we ran each of the four algorithms on the dataset five more times by removing one of the following groups of attributes: comorbidities, labs, radiology results, medication, and vital signs. The DT worked best without the vital signs, whereas the SVM and the logistic regression did not perform better without removing any of them. The relatively best result achieved was by removing comorbidities from the RF model which had 67% accuracy, 14% precision, and 47% recall (Table 4).

**Table 4:** Model performance.

Model name	Data	Precision	Recall	f1-score		Accuracy
				Not	Readmitted	
Decision tree	Before balancing	0.91	0.18	0.9	0.17	0.83
	After balancing	0.91	0.21	0.87	0.15	0.78
	Without comorbidities	0.91	0.16	0.88	0.13	0.8
	Without labs and radiology	0.91	0.2	0.89	0.16	0.8
	Without medications	0.91	0.2	0.89	0.17	0.81
	Without vitals	0.91	0.24	0.88	0.18	0.79
Support vector machine	Before balancing	0.9	0	0.95	0	0.9
	After balancing	0.91	0.04	0.94	0.04	0.89
	Without comorbidities	0.9	0	0.95	0	0.9
	Without labs and radiology	0.9	0	0.95	0	0.9
	Without medications	0.9	0	0.95	0	0.9
	Without vitals	0.9	0	0.95	0	0.9
Random forest	Before balancing	0.9	0	0.95	0	0.9
	After balancing	0.92	0.45	0.78	0.2	0.65
	Without comorbidities	0.93	0.47	0.79	0.21	0.67
	Without labs and radiology	0.92	0.45	0.78	0.2	0.66
	Without medications	0.92	0.4	0.82	0.2	0.7
	Without vitals	0.92	0.39	0.82	0.2	0.7
Logistic regression	Before balancing	0.9	0	0.95	0	0.9
	After balancing	0.9	0	0.95	0	0.9
	Without comorbidities	0.92	0.6	0.65	0.19	0.51
	Without labs and radiology	0.92	0.6	0.65	0.19	0.51
	Without medications	0.92	0.6	0.65	0.19	0.51
	Without vitals	0.92	0.6	0.65	0.19	0.51

## Discussion

Factors that affect patients’ readmission have been studied extensively in the past two decades. In this study, we studied clinical factors that can be linked to the risk of readmission for patients with CAP. The results of the analysis showed that patient age, heart rate, respiratory rate, medication count, and the number of comorbidities that the patient had were significantly associated with the odds of being readmitted. All other variables showed no significant effect. The significant variables from our analysis are all factors that could indicate the severity of the patient’s condition in general and during the initial admission.

However, when examining the magnitude of the relationship using odds ratios, we can observe two controversial themes. The first is that the worse patients have higher odds of readmission than their counterparts, which can be drawn from the odds of the temperature and number of comorbidities. The second theme is that worse patients have lower odds of being readmitted than their counterparts, which can be drawn from the odds of the age of the patient, heart rate, respiratory rate, and the number of medications prescribed. These two themes can be explained by the behavior of clinicians when dealing with admitted patients.

The researchers’ hypothesis is that factors such as age, heart rate, and respiratory rate are directly related to the condition of patients with CAP, whereas temperature and the number of comorbidities are not related. This implies that patients who are worse according to their age, heart rate, and respiratory rate should be given more attention and prescribed more medications, which subsequently results in lower odds of being readmitted. This explanation is consistent with another study conducted at the same hospital for studying seven-day readmission for emergency department patients [22]. Nevertheless, this hypothesis needs further investigation and likely an observational study to be confirmed or rejected.

To build a prediction model, multiple algorithms and techniques were available for use in this study. However, we selected the ones that can easily be interpreted by HCPs where each rule can be translated into if/then rules. Moreover, we included other models that have been showing promising results per our literature review. After extensive testing of the final clean dataset and multiple trials of creating the prediction model, although the accuracy of the model reached 90%, we did not find any appropriate prediction for readmission. This can be explained by the nature of our dataset because the data for readmitted patients within 30 days of discharge was only 9.1% of the dataset and not enough to create an appropriate prediction.

Looking at the results of the model, the SVM performed poorly, which contradicts the findings reported in the literature. In this study, SVM showed zero recall by predicting that all patients will not require readmission. Moreover, as part of the models’ training exercise, we tried to add and drop some of the predictors while using precision and recall to observe the effect on the performance of the models. This exercise resulted in a slight improvement in the RF model after decreasing the number of dummy attributes. On the other hand, better performance of the DT model was noticed after removing the continuous attributes of vital signs. Even with the best version of each model, we conclude that none of these models would qualify to serve as a reliable or valid model to predict readmissions of patients with CAP.

This poor performance of the models is in part due to the low quality of the dataset that was available for this analysis. For example, the laboratory results were provided as free text instead of structured numeric values, which required sophisticated and advanced text-mining efforts to extract the relevant values for our study. Unfortunately, this led to only including the number of labs that were ordered for each patient instead of the results of the labs. Conducting more natural language processing could have a real contribution in creating a model with better performance than our models.

Furthermore, in comorbidities, there were several entries for the same comorbidity for each patient. Therefore, the frequency of those comorbidities was not easily derived from the data. In addition, the same comorbidity had multiple descriptions which made it harder to calculate. For example, diabetes mellitus, type II diabetes, and type II diabetes mellitus can be found simultaneously, and they might have been documented differently for the same patient. Moreover, we used the International Statistical Classification of Diseases and Related Health Problems, Tenth Revision, Australian Modification codes of pneumonia for applying the inclusion criteria that only include patients who had CAP as a primary diagnosis. However, we noticed that different codes of pneumonia were used interchangeably in the HIS documentation even during the same visit, which affected the accuracy of including only CAP patients.

Dealing with such data requires a careful process to ensure that the cleaning process was done appropriately and did not result in faulty data or missing data from the original dataset. Furthermore, the decisions to choose how to transform the data should be discussed before each action with an expert in the field not only be taken by the data scientist. For example, because we had multiple readings of vital signs, we needed to transform it to get only one value for each visit; the data scientist might use the median value to give an overall view of the entire admission status, but the physician would rather take the last reading before discharge because it will better reveal how the patient was discharged, which will affect the readmission possibility. Another would rather use the worst reading during the admission to reflect how bad the patient’s condition was, which would affect the possibility of being readmitted.

The main limitation of this study was the structure of the original dataset, which limited the number of features that could be engineered and used for prediction. Another limitation was the lack of data outside the hospital that could strongly impact the readmission of patients. For example, if the hospital collected how well patients are adhering to their medications or their diet, we hypothesize that the prediction could have gained more accuracy. This kind of data could be collected as part of future studies from patients’ smartphones through dedicated applications. Future nationwide studies can be conducted to compare different populations based on different societal factors, such as lifestyle and diet, to determine their impact on readmission.

## Conclusions

Pneumonia is an infectious disease with major health and economic complications. Predicting CAP readmission using machine learning is an essential area of further research. Based on our study findings, the model accuracy reached 90%, but we did not find an appropriate prediction for readmission because the number of readmissions within 30 days was only 9.1% of the entire dataset received. We propose that machine learning can be used for predicting CAP readmission, but appropriate data sources and a suitable modeling technique are required to improve the accuracy of the model.

During the development of our machine learning and statistical analysis models, we identified factors that are associated with the readmission of patients with CAP. These factors should be used and closely monitored by HCPs to minimize the rate of readmissions of patients with CAP. In our study, we identified that less than 10% of the patients were readmitted for CAP after discharge within the dates included in our study. Furthermore, our results indicated that age, heart rate, respiratory rate, medication count, and the number of comorbidities that a patient had were significantly associated with the odds of being readmitted. However, the prediction performance of the models in our study was not adequate to predict the risk of readmission.

## References

[1] (2019). CDC. Pneumonia. https://www.cdc.gov/pneumonia/index.html.

[2] De Alba I, Amin A (2014). Pneumonia readmissions: risk factors and implications. Ochsner J.

[3] Capelastegui A, España Yandiola PP, Quintana JM (2009). Predictors of short-term rehospitalization following discharge of patients hospitalized with community-acquired pneumonia. Chest.

[4] Hebert C, Shivade C, Foraker R (2014). Diagnosis-specific readmission risk prediction using electronic health data: a retrospective cohort study. BMC Med Inform Decis Mak.

[5] Nagasako EM, Reidhead M, Waterman B, Dunagan WC (2014). Adding socioeconomic data to hospital readmissions calculations may produce more useful results. Health Aff (Millwood).

[6] Tang VL, Halm EA, Fine MJ, Johnson CS, Anzueto A, Mortensen EM (2014). Predictors of rehospitalization after admission for pneumonia in the veterans affairs healthcare system. J Hosp Med.

[7] Calvillo-King L, Arnold D, Eubank KJ, Lo M, Yunyongying P, Stieglitz H, Halm EA (2013). Impact of social factors on risk of readmission or mortality in pneumonia and heart failure: systematic review. J Gen Intern Med.

[8] Jencks SF, Williams MV, Coleman EA (2009). Rehospitalizations among patients in the Medicare fee-for-service program. N Engl J Med.

[9] Epstein AM, Jha AK, Orav EJ (2011). The relationship between hospital admission rates and rehospitalizations. N Engl J Med.

[10] Zou Q, Qu K, Luo Y, Yin D, Ju Y, Tang H (2018). Predicting diabetes mellitus with machine learning techniques. Front Genet.

[11] Act AC (200). Patient Protection and Affordable Care Act. https://www.connectthedotsusa.com/wp-content/uploads/2019/06/ACAvNIMASlidesScript_6_3_19.pdf.

[12] Mather JF, Fortunato GJ, Ash JL, Davis MJ, Kumar A (2014). Prediction of pneumonia 30-day readmissions: a single-center attempt to increase model performance. Respir Care.

[13] Weinreich M, Nguyen OK, Wang D, Mayo H, Mortensen EM, Halm EA, Makam AN (2016). Predicting the risk of readmission in pneumonia. A systematic review of model performance. Ann Am Thorac Soc.

[14] O'Brien WJ, Chen Q, Mull HJ, Shwartz M, Borzecki AM, Hanchate A, Rosen AK (2015). What is the value of adding Medicare data in estimating VA hospital readmission rates?. Health Serv Res.

[15] Al Ghamdi H, Alshammari R (2017). Predicting hospital readmission within thirty-days. J Med Imaging Health Inform.

[16] Pedregosa F, Varoquaux G, Gramfort A (2011). Scikit-learn: machine learning in Python. J Mach Learn Res.

[17] Breiman L (2001). Random forests. Mach Learn.

[18] Fan RE, Chang KW, Hsieh CJ, Wang XR, Lin CJ: LIBLINEAR (2008). LIBLINEAR: a library for large linear classification. J Mach Learn Res.

[19] Quinlan JR (1986). Induction of decision trees. Mach Learn.

[20] Cortes C, Vapnik V (1995). Support-vector networks. Mach Learn.

[21] Chawla NV, Bowyer KW, Hall LO, Kegelmeyer WP (2002). SMOTE: synthetic minority over-sampling technique. J Artif Intell Res.

[22] Aldhoayan MD, Khayat AM (2022). Leveraging advanced data analytics to predict the risk of all-cause seven-day emergency readmissions. Cureus.

